# Whole genome comparative analysis shows limited *Escherichia coli* co-occurrence within co-habiting humans and dogs

**DOI:** 10.1186/s12864-026-13236-2

**Published:** 2026-08-04

**Authors:** Gabriel Astorga, Samuel J. Bloomfield, John Wain, Leonardo de Oliveira Martins, Dipali Singh, Sumeet K. Tiwari, George M. Savva, Anette Loeffler, Juliana Menezes, Constança Pomba, Siân Marie Frosini, Alison E. Mather

**Affiliations:** 1 Quadram Institute Bioscience, Norwich Research Park, Norwich, NR4 7UQ UK; 2 Centre for Microbial Interactions, Norwich Research Park, Norwich, NR4 7UG UK; 3 Department of Clinical Science and Services, Royal Veterinary College, Hertfordshire, Hatfield, AL9 7TA UK; 4 Department of Pathobiology and Population Sciences, Royal Veterinary College, Hertfordshire, Hatfield, AL9 7TA UK; 5 Centre for Interdisciplinary Research in Animal Health (CIISA), Faculty of Veterinary Medicine, University of Lisbon, Lisbon, Portugal; 6 Associate Laboratory for Animal and Veterinary Sciences (AL4AnimalS), Lisbon, Portugal; 7 I-MVET, Research in Veterinary Medicine, Faculty of Veterinary Medicine, Lusófona University - Lisbon University Centre, Lisbon, Portugal; 8 University of East Anglia, Norwich Research Park, Norwich, NR4 7TJ UK; 9 Department of Electrical Engineering and Electronics, University of Liverpool, Liverpool, L69 7ZF UK

**Keywords:** *Escherichia coli*, Genome-Wide Association Study, Antimicrobial Resistance, Multidrug Resistance, One Health, Companion Animals

## Abstract

**Background:**

Dogs are one of the most popular companion animals in the UK, with an estimated 13.5 million within UK households. Within these households, human-animal physical interactions may lead to inter-species bacterial sharing, including *Escherichia coli*. *E. coli* is an opportunistic pathogen with a broad host range and can contribute to the spread of antimicrobial resistance (AMR). This work aimed to understand the extent of *E. coli* co-occurrence between hosts within the same household, and between dogs and humans.

**Results:**

*E. coli* was non-selectively cultured from faecal samples obtained from 24 households with at least one human (*n* = 31) and one dog (*n* = 27) sample available at the same timepoint. Up to eight presumptive *E. coli* colonies were selected per sample and underwent short read whole genome sequencing. In total, 217 *E. coli* genomes were classified into 53 known sequence types (STs) and one unknown ST. Within samples, up to four different STs were present within each human and up to three different STs within a dog. Using a five single nucleotide polymorphism (SNP) threshold, there were 20 *E. coli* co-occurrences between hosts, including the same and different host species. Four instances involved potential cross-species co-occurrence within the same household. A genome-wide association study using a combined unitig and gene presence and absence approach identified one gene significantly more common in human derived samples; in silico characterisation revealed this gene is likely to have a function related to Type-F conjugative transfer system pilin assembly. Overall, 48.8% of the genomes had at least one genetic determinant associated with AMR, and 17.5% were classified as multidrug resistant. *E. coli* from humans carried more AMR determinants than *E. coli* from dogs.

**Conclusion:**

There was limited evidence of *E. coli* co-occurrence between different host species within households, and only one host-associated genetic feature. Humans harboured *E. coli* associated with more AMR determinants than those from dogs, suggesting that *E. coli* from humans presented a larger AMR source and AMR risk to other hosts in this dataset. Our findings can help inform risk analysis of *E. coli* sharing and transmission between humans and their companion dogs.

**Supplementary Information:**

The online version contains supplementary material available at https://doi.org/10.1186/s12864-026-13236-2.

## Background

In 2024, one of the most popular domesticated companion animals in UK households was the pet dog [59], *Canis lupus familiaris*. In the UK there is estimated to be 13.5 million dogs kept as pets, with 36% of households having a dog [85]. The shared environment and lifestyles of humans and companion dogs can affect daily habits and the microbial exposure of both hosts [20]. *Escherichia coli* is a common gut coloniser [26], and the human gut harbours a mixture of different types of residential and transient *E. coli* [26, 72]. *E. coli* can be found within both humans and dogs and has been suggested to successfully colonise both hosts because of repeated exposure and cohabitation [34, 35].

*E. coli* can carry genetic determinants conferring antimicrobial resistance (AMR) and is often used as an indicator organism to assess AMR risk [50, 68]. AMR is a major global threat, as medically important antimicrobials may become less effective for treating bacterial infections, leading to longer and more complex treatments, and higher mortality rates, [60, 91]. In 2019, AMR was estimated to be involved in 4.95 million deaths globally with antimicrobial resistant *E. coli* being one of the main organisms involved [54].

Identifying bacterial transmission (indicating direction) and the lesser used term sharing (unknown directionality) between hosts requires longitudinal and thorough epidemiological data. For cross-sectional studies, potential transmission or sharing cannot be definitively determined, meaning that observed genetically similar *E. coli* should be interpreted as co-occurrence. Human to human *E. coli* transmission has been documented in nosocomial settings using a longitudinal study [46], as well as the co-presence of indistinguishable *E. coli* between humans within the same household [36].

Humans and companion dogs can be asymptomatic carriers of similar *E. coli* as determined by identical pulsed-field gel electrophoresis (PFGE) results, AMR phenotypes and genotypes, with potential sharing observed [28, 30, 55, 69, 78, 84, 87, 89]. PFGE approaches lack the resolution to identify genetically clonal *E. coli*. Whole genome sequencing (WGS) resolves this as single nucleotide differences can be identified. Genetically similar *E. coli*, defined as *E. coli* with a low number of single nucleotide polymorphism (SNP) differences (1–2 SNPs), with the same AMR determinants have been found in both humans and companion dogs from the same household [22, 50]. WGS has been used to identify *E. coli* co-carriage in humans and dogs, but there has been a focus on selecting for AMR phenotypes [19, 50, 87]. Selecting for AMR *E. coli* limits understanding into potential sharing of the broader microbial population. For this, a non-antimicrobial-selective WGS approach is needed to assess the genetic similarity between commensal *E. coli* within humans and companion dogs and potential AMR risk to hosts.

The main aims of this study were to: 1) Use a non-antimicrobial-selective WGS approach to investigate the co-occurrence of closely related *E. coli* within and between humans and companion dogs; 2) Compare *E. coli* genomes from humans and dogs to identify genetic similarities and differences within and between hosts and households; 3) Assess AMR determinants and extra-intestinal pathogenic potential of *E. coli* from different hosts; and 4) Assess the risk to humans and dogs from potential co-occurrence of *E. coli.*

## Methods

### Human and companion dog study population

Faecal samples were collected following signed, informed consent prior to enrolment as part of a previously described international longitudinal observational study at the Royal Veterinary College (RVC), Hertfordshire, UK [50] with ethical approval from the RVC’s Clinical Research Ethical Review Board (CRERB; URN 2017 1750–3). As described in previous work, this study included UK households with at least one human and at least one companion dog living in close contact using convenience sampling.

An epidemiological questionnaire was completed by human members of each household [50]. Companion animals’ health was evaluated by veterinary professionals, supplemented by behavioural data provided by the owner.

### Sample collection and processing

The human and animal faecal sample collection process is described in previous work by Menezes et al. [50], and briefly summarised in supplementary methods S1. Faecal samples were stored in 0.9% NaCl (Merck, Germany) and 50% glycerol (Sigma-Aldrich, USA) in the RVC biobank at −70/−80 °C.

Faecal samples were obtained as convenience samples and varied in initial mass, which was not recorded. One faecal sample from each host was defrosted, homogenised then enriched in a 1:1 ratio of buffered peptone water (Biokar Diagnostics, France). Enriched samples were incubated at 37 °C for 18 ± 4 h. All subsequent incubations were performed at 37 °C for 18 ± 4 h. For each enriched sample, a 1:10 dilution was performed using 0.9% saline, and 100 μl of the enrichment and dilution were plated on Tryptone Bile X-Glucuronide (TBX, Oxoid, Basingstoke, UK) agar and incubated.

Up to eight presumptive *E. coli* colonies from each TBX agar plate (further details are described in supplementary methods S2) were streaked onto 5% sheep’s blood agar (Thermo Fisher Scientific, Basingstoke, UK) and incubated. Colonies were biochemically confirmed as presumptive *E. coli* if they were citrate negative using Simmons’ citrate agar (Sigma-Aldrich, Haverhill, UK) and indole positive using Kovac’s indole spot reagent test (Fisher Scientific, Loughborough, UK). Biochemically confirmed colonies were streaked onto Mueller–Hinton agar, incubated, then transported on Amies transport swabs (Scientific Laboratory Supplies, Nottingham, UK) to Quadram Institute Bioscience. Each colony was subcultured onto tryptic soy agar (Trafalgar Scientific Ltd., Leicester, UK).

The association between human and dog *E. coli* carriage was done at the household level. A host was classified as *E. coli*-positive if the faecal sample had *E. coli* present. Households without samples from both humans and dogs were excluded. The odds ratio was calculated using a 2 × 2 contingency table, and the 95% confidence intervals and p-values were calculated using the Fisher’s Exact test in R v4.1.3 [42].

The households from the previously described international longitudinal observational study at the Royal Veterinary College (RVC), Hertfordshire, UK [50] were sampled longitudinally. A “timepoint” was defined as a time when faecal samples were provided from both humans and dogs. The analysis was restricted to the first timepoint of a household that met the criteria outlined below. Therefore, the work within this study did not explore within-household co-occurrence over time.

A household was termed “complete” according to the following inclusion criteria: (i) at least one healthy human and at least one healthy dog cohabiting, to reflect a typical household and avoid healthcare-associated biases on *E. coli* carriage; (ii) faecal samples where there was at least one sample available from the same timepoint from both humans and dogs within the same household; (iii) at least one *E. coli* isolated from at least one healthy human and one healthy dog in a household.

### Whole genome sequencing, quality control and assembly

*E. coli* DNA was extracted using the Promega Maxwell RSC Cultured Cell DNA kits (Promega, Southampton, UK) according to manufacturer instructions. Libraries were prepared using Nextera XT library preparation kits (Illumina, San Diego, California, USA) and sequenced on an Illumina NextSeq system, creating 150 bp paired-end reads. Post WGS, isolated *E. coli* colonies are referred to as genomes. Genomes were analysed on a high-performance computing (HPC) cluster at the Norwich Bioscience Institutes and on the Quadram Institute Bioscience QIB-CLOUD Virtual Machine. Genomes were deposited in the European Nucleotide Archive under the accession ID PRJEB104018.

Adapter sequences were trimmed using Trimmomatic v0.33 (parameters stated in Supplementary Methods S3) [11], assembled into contiguous sequences using SPAdes v3.1.1 with the ‘-careful’ parameter and with k-mer lengths set to 21, 33, 55 and 77 [5]. Assembly quality was analysed using QUAST v4.6.3 [27], CheckM v1.0.18 using default parameters, and by aligning the trimmed reads to the genome assemblies using the Burrows-Wheeler aligner v0.7.17 [44], and analysing the read depth and coverage using samtools v1.9 [45] and bcftools v18 [23] using default parameters.

*E. coli* genomes passed quality control if they were classified as *Escherichia coli* by Centrifuge v1.0.3 [41], had less than 50 duplicate genes, were 4.4 Mbp – 5.7 Mbp in length, had an average GC content between 42.6%−54.6% [25], and had a mean read depth of their four largest contigs above 28, which is sufficient for reliable SNP calling and core-genome typing [10, 15].

### *E. coli* phylogroup, sequence typing, and phylogenetic analysis

Assembled *E. coli* genomes were characterised into phylogroups using Clermontyping v20.03 [6]; the Clermontyping scheme is based on the presence and absence of the genetic markers *arpA, chuA, yjaA*, TspE4.C2, phylogroup E and C specific markers (Gp.E and Gp.C, respectively), and *Escherichia* cryptic clade specific markers (Cl.I, Cl.II, Cl.III, Cl.IV and Cl.V). Multi-locus sequence types (STs) were determined using MLST v2.19.0 [76] with the *E. coli* pubMLST seven gene scheme (https://github.com/tseemann/mlst). Unique combinations of *adk*, *fumC*, *gyrB*, *icd*, *mdh*, *purA*, and *recA* profiles provided by the MLST output were used to characterise unclassified STs into matching novel STs. Serogroups were determined using ECtyper 2.0.0 using default parameters on assemblies [9].

Phylonium v1.6 [42] with default settings was used to estimate the phylogenetic distances between the *E. coli* assemblies. A dendrogram was estimated using rapidNJ v2.3.2 [77] and was visualised using R v4.1.3 [63] and ggtree v3.10.1 [93]. Bootstrap support values were calculated using the -b = 1000 parameter in Phylonium v1.6, constructing neighbour-joining trees with RapidNJ v2.3.2, and summarising support values across replicates using SumTrees programme as part of DendroPy v5.0.8 [80].

### Antimicrobial resistance determinants and virulence genes

The presence of AMR genes, plasmid replicons and virulence genes were determined using ARIBA v2.14.6 [32] with default settings on the trimmed reads, with the Resfinder v4.3.1 [12], PlasmidFinder v2.1.0 [16], and VFDB (virulence factor database) core [17] databases, respectively, which were all downloaded in May 2023. Known point mutations associated with AMR were identified using PointFinder (python script downloaded in October 2024) (https://github.com/guthrielab/pointfinder) [94]. AMR genes and point mutations were collectively termed AMR determinants.

The AMR determinants present were categorised into the antimicrobial classes to which they conferred resistance and priority according to the World Health Organization scheme [92]. Putative multidrug-resistance (MDR) in this study was classified as the presence of AMR genes or point mutations conferring resistance to three or more different antimicrobial classes according to the WHO antimicrobial class list [47, 91].

*E. coli* genomes classified as the same ST with the same AMR determinant combination (termed AMR genotypes) from each individual sample were de-replicated. This de-replicated ST-AMR dataset was used to identify the number of unique AMR genotypes. The Wilcoxon rank-sum test was used to assess whether or not *E. coli* populations from different hosts (humans and dogs) differed in the number of unique AMR determinants per ST-AMR genotype.

*E. coli* isolates were characterised as extraintestinal pathogenic (ExPEC) lineages if two or more of the following virulence genes were present in the genome: *papAHC, sfa/focDE, afa/draBC, iutA,* and *kpsMII* [37].

### Pairwise SNP analysis

Genomes were annotated using Prokka v1.13.3 [73] and GFF files were used to create a core gene alignment (genes present in 95% of genomes; minimum percent identity set to 90%) using Roary v3.12.0 [57]. Pairwise SNP differences between isolates were identified by using snp-dists v0.7 [75]. The threshold to determine the closely related isolates within the same sample was five SNPs, the UKHSA threshold for an outbreak of Shiga toxin producing *E. coli* O157 [51]. *E. coli* STs identified in different hosts with less than five SNP differences using this gene alignment approach were taken forward for a reference-based SNP analysis approach. Additionally, *E. coli* STs present in both humans and dogs were chosen for further reference-based SNP analysis.

A reference genome for each selected ST was chosen using Referenceseeker v1.8.0 [70] and the bacteria RefSeq database (downloaded January 2024) [56]. The rationale for choosing the reference for each ST is stated in Supplementary methods S4.

The presence of putative *E. coli* clones in different hosts was investigated. A reference-based core genome alignment approach was used for each examined ST as it provides high resolution insight and consistent nucleotide positional information into SNP differences. To optimise a tree-like structure, estimated regions of recombination were removed from the genomes as these regions would inflate genetic distances between closely related *E. coli*. Reads for each ST were aligned to their respective reference genome using Snippy v4.6.0 [74]. SNPs attributed to putative recombination from the alignments were removed using Gubbins v3.2.0. For each ST, the pairwise SNP differences were calculated using the reference-based core genome alignments with snp-dists v0.7. The UKHSA threshold for an outbreak of Shiga toxin producing *E. coli* O157 [51] was also applied to this reference-based analysis. Genomes with five or less SNPs with the same combination of AMR determinants were defined as putative clones and the presence of putative *E. coli* clones within different individuals was defined as a co-occurrence.

In this work, *fimH* alleles were determined using Fimtyper v1.0 under the Center for Genomic Epidemiology (CGE) server (https://www.genomicepidemiology.org; accessed 25/06/2025) [66].

### Genome-wide association analysis

The genome-wide association analyses were performed on the entire dataset using a combined unitig and gene presence and absence approach to identify genes associated with host using Pyseer v1.3.10 [43].

For the unitig approach, unitigs were identified from assemblies using unitig-caller v1.3.0 [31]. Unitigs associated with the host phenotype (human or dog) were identified using a linear mixed model approach. Using Pyseer, the -lmm mode was used with the -distances parameter and a distance matrix estimated by Phylonium v1.6 to correct for population structure. A covariate matrix consisting of household information was used as input for the -covariates parameter and accounted for household as a potential confounding factor.

To correct for population structure, patristic distances were generated from the Phylonium output and incorporated into the model using the Pyseer v1.3.10 phylogeny_distance.py For the gene presence and absence approach, Roary v3.12.0 [57] was used to form a gene presence and absence matrix using previously stated parameters. For each gene in the accessory genome, a logistic regression model was used to investigate associations between the host (human or dog) using Pyseer with the -lmm approach script; the distance matrix was used as input for the -distances parameter. The maximum number of dimensions was calculated using the Pyseer v1.3.10 scree_plot_pyseer command, and the number of dimensions was selected as three using the -max-dimensions parameter according to developer recommendations. A covariate matrix consisting of household information was used as input for the -covariates parameter.

Unitigs and genes were pre-filtered using Pyseer default parameters. The threshold for significantly associated unitigs and genes was determined for both approaches. The Pyseer -output-patterns parameter was used to generate separate files with hashes corresponding to the unique presence and absence pattern of each unitig and each gene. Using the Pyseer v1.3.10 count_patterns.py script, the number of unique patterns was calculated for both approaches. As part of the Pyseer workflow, a Bonferroni correction was applied to determine the significance threshold for both unitigs and genes, which was calculated separately for the two approaches by dividing 0.05 by the respective total number of unique patterns. Unitigs and genes were considered significantly associated if their respective p-values were lower than the respective Bonferroni-corrected significance threshold.

To assess the confidence in the GWAS results a permutation test was used. Both the unitig and the gene presence and absence approach were applied to the same 10 datasets where the host phenotype (human and dog) associated with each genome was randomly shuffled. The host phenotype associated with each genome was randomised with replacement using the “shuf” command in Linux and the number of genomes was kept consistent. The randomised datasets were analysed using the same parameters as the original analysis.

The candidate hypothetical proteins classified as significantly associated with host species using both approaches were queried against the NCBI nr database [date accessed: 19/09/2024] using BLASTP v2.16.1 + [2] with default parameters.

### Putative plasmid contigs

Putative plasmid contigs were predicted using RFplasmid v1.0 using the *E. coli* species model [88]. Contigs with a plasmid vote ≥ 0.6 were extracted from assemblies. AMR genes found on these putative plasmid contigs were identified using Abricate v0.7 (https://github.com/tseemann/ABRICATE) with the Resfinder database using the July 16th 2018 database. A subset of isolates (n = 9) was additionally sequenced using long-read technology to enable plasmid resolution (see Supplementary Methods S5). Long read sequences were deposited in the European Nucleotide Archive under the accession ID PRJEB104018.

## Results

**Fig. 1:**
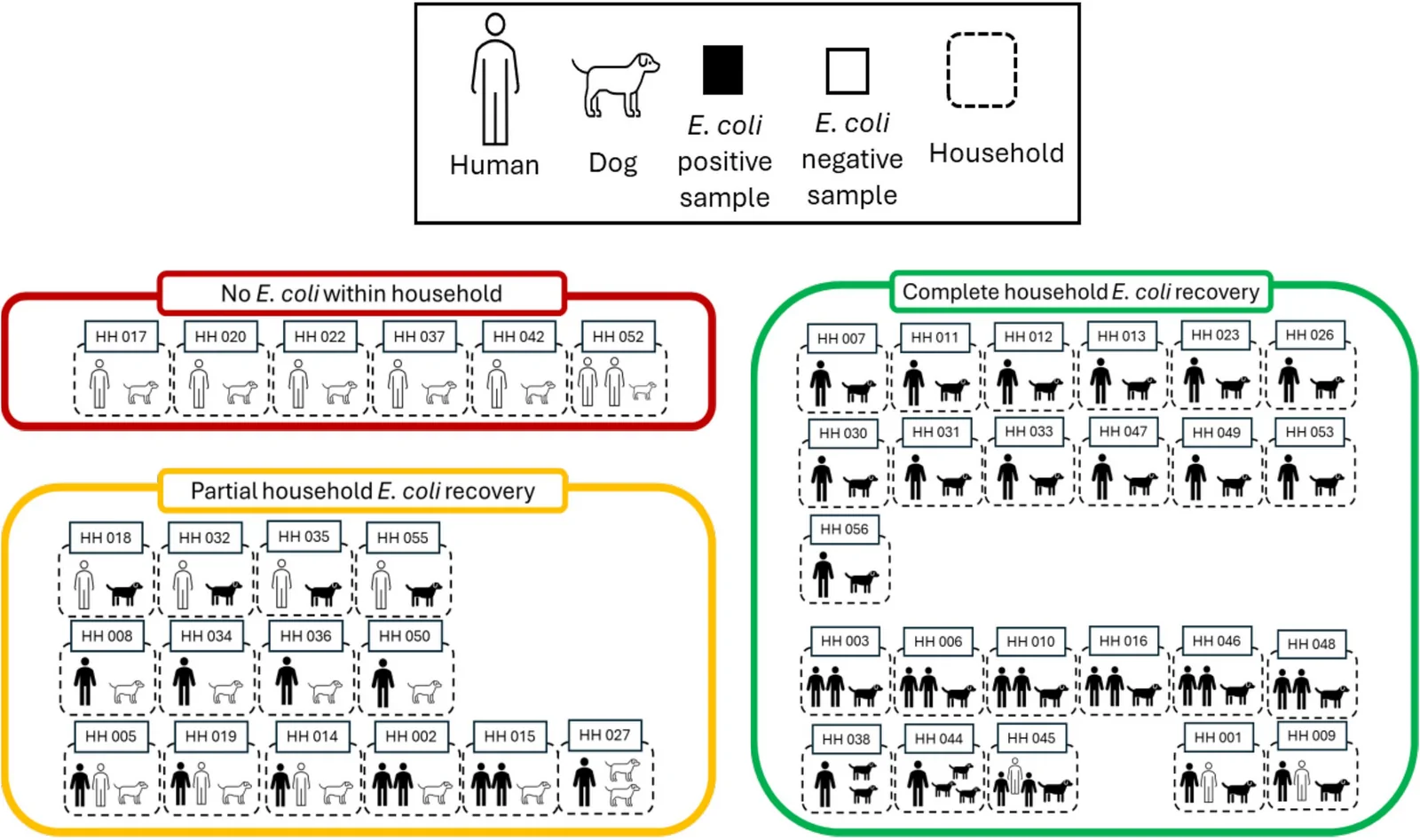
*E. coli* recovery from faecal samples from 44 dog-owning households, separated by absence, partial presence and complete households, where at least one human and one dog had *E. coli* recovered from their previously collected faecal samples. Households (HH) are denoted by dotted lines and given an identifier number. Hosts with *E. coli* positive faecal samples are represented by a filled silhouette

From the initial 56 households, 10 households were excluded as the dogs were not healthy. Additionally, two households only had faecal samples from one host species and were therefore not analysed. A total of 44 UK households with at least one human and at least one companion dog living in close contact were selected for this study. In total, 24 households were termed “complete” and had at least one human (total number of humans = 31) and at least one dog (total number of dogs = 27) where at least one *E. coli* colony was recovered (Fig. 1) at the same timepoint. The samples in this work were collected between February 2018 – January 2019 (Supplementary Table 1).

### Study population and *E. coli* recovery

*E. coli* was recovered from 74/108 (68.5%) of all samples. *E. coli* was culturable from 43/60 (71.7%) human samples and 31/48 (64.6%) dog samples. Only *E. coli* from complete households were used for downstream analysis. The odds of human faecal samples with positive *E. coli* carriage were higher in households with dogs also having *E. coli* carriage, but the association was not statistically significant due to the small sample size (OR = 3.49, 95% CI = 0.662—20.8, *p* = 0.133).

All humans from complete households were at least 18 years old, with 67.7% (*n* = 21) above 40 years old. All companion dogs from complete households had metadata and signalment (animal information including breeds, ages and sex), except for one dog which had no age, weight and sex data available. The dog ages ranged from eight months to 15 years and nine months (median = seven years and nine months). Approximate weights ranged from 5 to 35 kg (median = 14.5 kg), 50.0% (*n* = 13) were male, and 50.0% (*n* = 13) were female (Supplementary Table S0).

### Phylogroups and STs

**Fig. 2:**
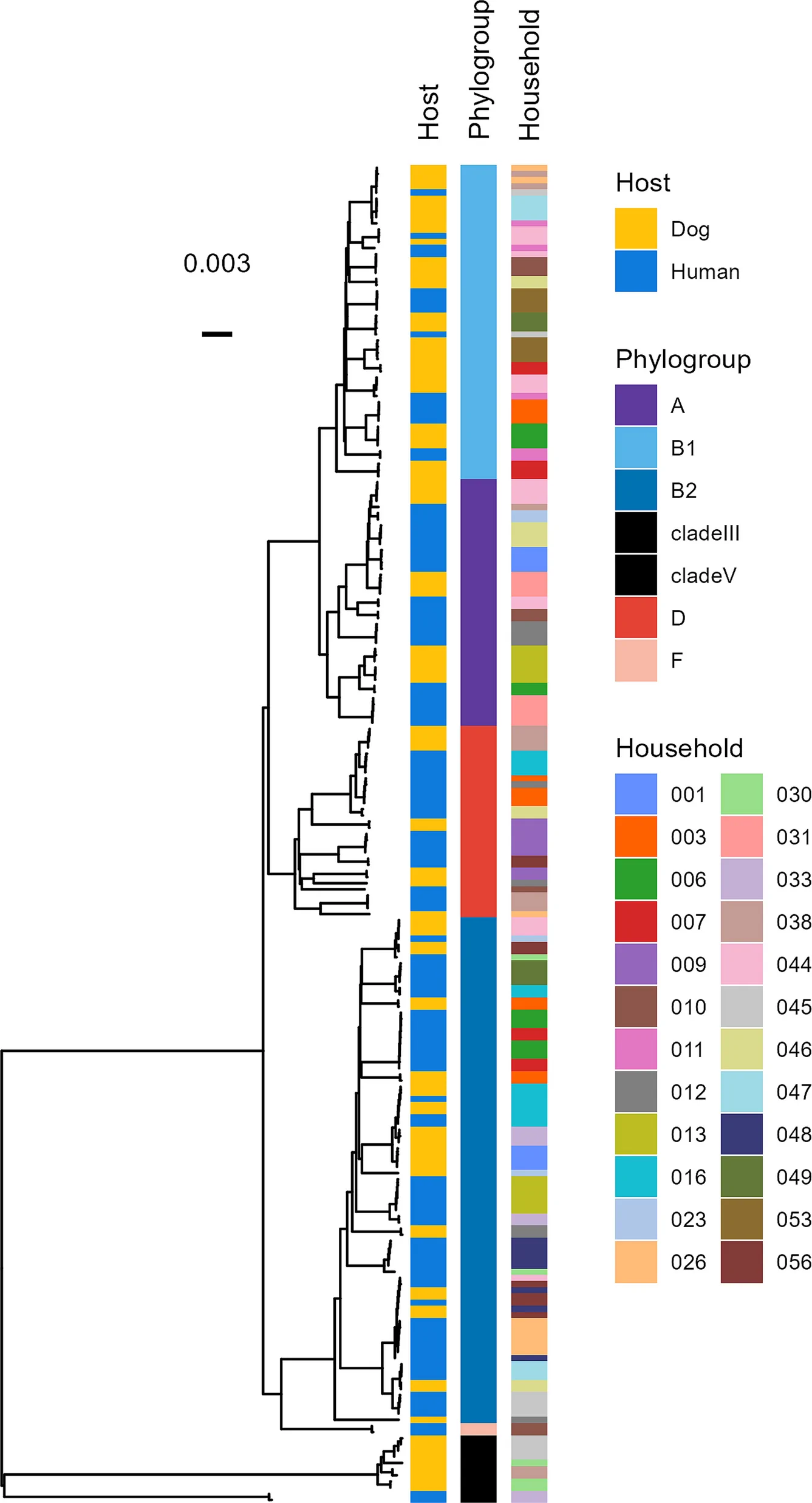
Phylonium midpoint rooted phylogenetic tree of the collection of 217 *E. coli* genomes isolated from humans and their companion dogs from 24 households, with a tree scale estimating 0.003 nucleotide substitutions per site. The host species (left column), phylogroup (middle column) and household (right column) are plotted against the tree

The 217 *E. coli* genomes were classified into four predominant phylogroups—B2 (37.8%), B1 (23.5%), A (18.4%), and D (14.3%) (Fig. 2; Supplementary Fig. 2), with phylogroup F and cryptic clades III and V also identified. The genomes were classified into 53 known STs and one novel ST (Supplementary Table S1 and S2), the most common classification being ST131 (10.6%, n genomes = 23) (Supplementary Table S1). Within this dataset, 12/31 (38.7%) humans and 12/27 (44.4%) dogs harboured multiple STs. A maximum of four STs were identified in an individual human sample, whereas a maximum of three STs were identified in an individual dog sample (Table 1). Nine STs were found in both humans and dogs – ST2163, ST101, ST12, ST10, ST372, ST1079, ST131, ST1800, and ST164 (Supplementary Fig. 3). The serogroups of all *E. coli* isolates were identified from the genomes and are summarised in Supplementary Table S1.

**Table 1:** Unique *E. coli* sequence types (STs) identified in faecal samples from humans (H) and their companion dogs (D) from 24 households (HH). The novel ST is defined in Supplementary Table S2

Household	Host	Number of isolates taken	Unique ST
**HH 001**	**D1**	**4**	**961**
	**H1**	**4**	**8125**
**HH 003**	**D1**	**4**	**978, 1442**
	**H1**	**5**	**69**
	**H2**	**4**	**3759**
**HH 006**	**D1**	**4**	**3576**
	**H1**	**4**	**141**
	**H2**	**4**	**141, 635**
**HH 007**	**D1**	**5**	**642, Novel1**
	**H1**	**4**	**141**
**HH 009**	**D1**	**4**	**720, 8573**
	**H1**	**4**	**38**
**HH 010**	**D1**	**3**	**2163**
	**H1**	**4**	**59, 4429**
	**H2**	**1**	**362**
**HH 011**	**D1**	**1**	**101**
	**H1**	**4**	**906, 1079**
**HH 012**	**D1**	**4**	**589, 2538, 5254**
	**H1**	**4**	**710**
**HH 013**	**D1**	**6**	**399, 7075**
	**H1**	**6**	**95**
**HH 016**	**D1**	**4**	**12**
	**H1**	**4**	**9265**
	**H2**	**5**	**12, 73**
**HH 023**	**D1**	**1**	**6164**
	**H1**	**3**	**10, 372**
**HH 026**	**D1**	**3**	**101, 349**
	**H1**	**6**	**131**
**HH 030**	**D1**	**3**	**7345, 7347**
	**H1**	**2**	**372, 404**
**HH 031**	**D1**	**4**	**10**
	**H1**	**5**	**5295**
**HH 033**	**D1**	**3**	**12**
	**H1**	**4**	**95, 5345**
**HH 038**	**D1**	**4**	**106**
	**D2**	**4**	**101, 2559**
	**H1**	**4**	**10, 70**
**HH 044**	**D1**	**4**	**372, 1079**
	**D2**	**4**	**10**
	**D3**	**4**	**1079, 164**
	**H1**	**5**	**131, 164, 542, 2163**
**HH 045**	**D1**	**4**	**7236**
	**H1**	**4**	**131**
	**H3**	**2**	**101, 1800**
**HH 046**	**D1**	**4**	**131, 942**
	**H1**	**4**	**34**
	**H2**	**2**	**69**
**HH 047**	**D1**	**4**	**101**
	**H1**	**3**	**131**
**HH 048**	**D1**	**2**	**131**
	**H1**	**2**	**131, 1193**
	**H2**	**4**	**1193**
**HH 049**	**D1**	**3**	**1800**
	**H1**	**4**	**73**
**HH 053**	**D1**	**4**	**5415**
	**H1**	**4**	**58**
**HH 056**	**D1**	**4**	**131, 372**
	**H1**	**4**	**131, 963**

### Genome-wide association study

The Bonferroni-corrected significance threshold for unitigs was 7.45 × 10^–8^. A total of 1,802,136 variants were evaluated and pre-filtered using the Pyseer workflow. After pre-filtering, 1,517,048 were tested for association with the host phenotype. Unitig GWAS analysis identified 142 unitigs associated with host species. These unitigs mapped to 45 genes and contig IDs. In this analysis, 12 contig IDs associated with humans and 14 contig IDs associated with dogs did not directly map to an annotated gene (p-values reported in Supplementary Table S3).

The Bonferroni-corrected significance threshold for the gene presence and absence approach was 7.53 × 10^–6^. After evaluating and pre-filtering 25,451 gene presence and absence clusters of orthologous genes, 15,006 passed pre-filtering and were tested for association with the host phenotype. The gene presence and absence approach identified 22 clusters of orthologous genes associated with human hosts and 19 associated with dog hosts (p-values reported in Supplementary Table S4).

**Fig. 3:**
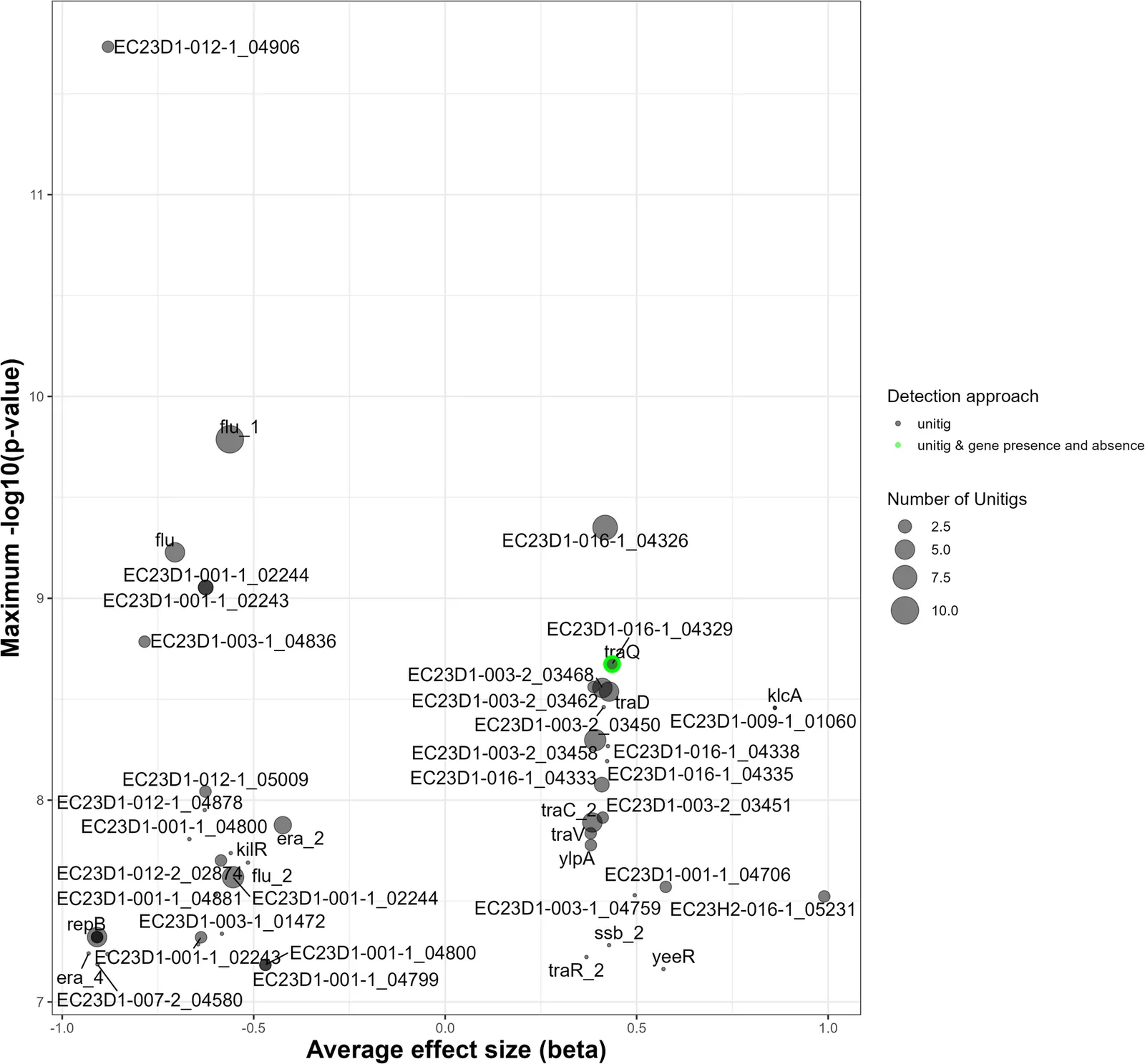
The unitig-based genome-wide association results showing significantly associated *E. coli* genes and hypothetical genes with humans and dogs. The X axis indicates the average effect size (beta) value, where a higher beta value indicates an association with human source, and a lower beta value indicates an association with dog source. The Y-axis shows the maximum –log10(p-value) among those k-mers, where higher values indicate stronger evidence of association. The size of the bubble indicates the number of significant unitigs mapping onto the gene. Labels starting with “EC23” indicate coding DNA sequences that lack a functional annotation and are labelled by their Pyseer identifier (e.g., EC23D1-016-1_04329). The single significantly host-associated gene using both the unitig and pangenome gene presence and absence approach is highlighted in green

However, there was little overlap between the two approaches. Using results from both the gene presence and absence approach and the unitig approach, one gene was identified as significantly associated with humans using both methods. The human-associated gene was a hypothetical protein, which was identified as the gene ID EC23D1-016-1_04329|TraQ and mapped onto the hypothetical protein group 4813 (Fig. 3). The datasets where the host phenotype (human vs dog) associated with each genome were randomised had no significantly associated unitigs or genes.

BLASTn analysis identified a 100% query cover and 100% percent identity (E value = 1e-130) similarity between the gene associated with human host and a type-F conjugative transfer system pilin assembly thiol-disulphide isomerase (TrbB) (Accession number: WP_000059831.1). The gene was found in 47 *E. coli* genomes, including 41 from humans (n samples = 13) and six from dogs (n samples = 2). *E. coli* genomes with this gene were found in phylogroups A, B2 and D (Supplementary Fig. 4).

RFplasmid detected 1,674 putative plasmid contigs in this dataset. The two unitig sequences associated with the human-associated gene *trbB* were found within 22 plasmid contigs from 22 different genomes belonging to six STs (Supplementary Table S5; Table S6). Of these 22 genomes, 18 contained *trbB*. Plasmid contigs with the human-associated unitigs present were found in five ST12 genomes from the same household from both the human and companion dog. One of these ST12 genomes from a human sample had a 68,569 bp putative plasmid contig with the *trbB*-human-associated unitigs, as well as antimicrobial resistance genes *tet*(B) and *bla*_TEM-1B_.

Long-read sequencing of a subset of isolates that harboured *trbB* on plasmid contigs with the IncFIB(AP001918)_1 plasmid replicon (n = 9) was performed. In these genomes, the *trbB*-containing plasmids contained 0–3 AMR genes and these AMR genes were found in close proximity to insertion sequences in a specific region of this plasmid (see Supplementary Results R1; Supplementary Fig. 5).

### Antimicrobial resistance determinants and putative ExPEC

In this dataset, 106 of 217 (48.8%) of *E. coli* genomes contained at least one AMR determinant (Supplementary Table S1). Six samples contained isolates that belonged to the same ST but had different AMR genotypes. A maximum of three unique AMR genotypes were identified in one human sample that had four ST710 *E. coli*. A maximum of two unique AMR genotypes were identified in *E. coli* from two dog samples where more than one genome was classified as the same ST10 or ST12 (Supplementary Table S7).

Within this collection, 38 (17.5%) *E. coli* genomes from humans (31 genomes from 13 samples) and dogs (7 genomes from 3 samples) had putative multi-drug resistance genotypes.

Virulence gene analysis revealed no *stx* genes, while the *eae* fimbriae gene was identified in two *E. coli* genomes from the same dog sample. There were 19/217 (8.76%) genomes across eight samples (n human samples = 6; n dog samples = 2) classified as putative ExPEC (Supplementary Table S1). These genomes belonged to eight ST95 (3.69%), five ST372 (2.30%), four ST9265 (1.84%), and two ST73 (0.922%). All eight samples containing putative ExPEC were from different households.

### Co-occurrence within and between households

**Fig. 4:**
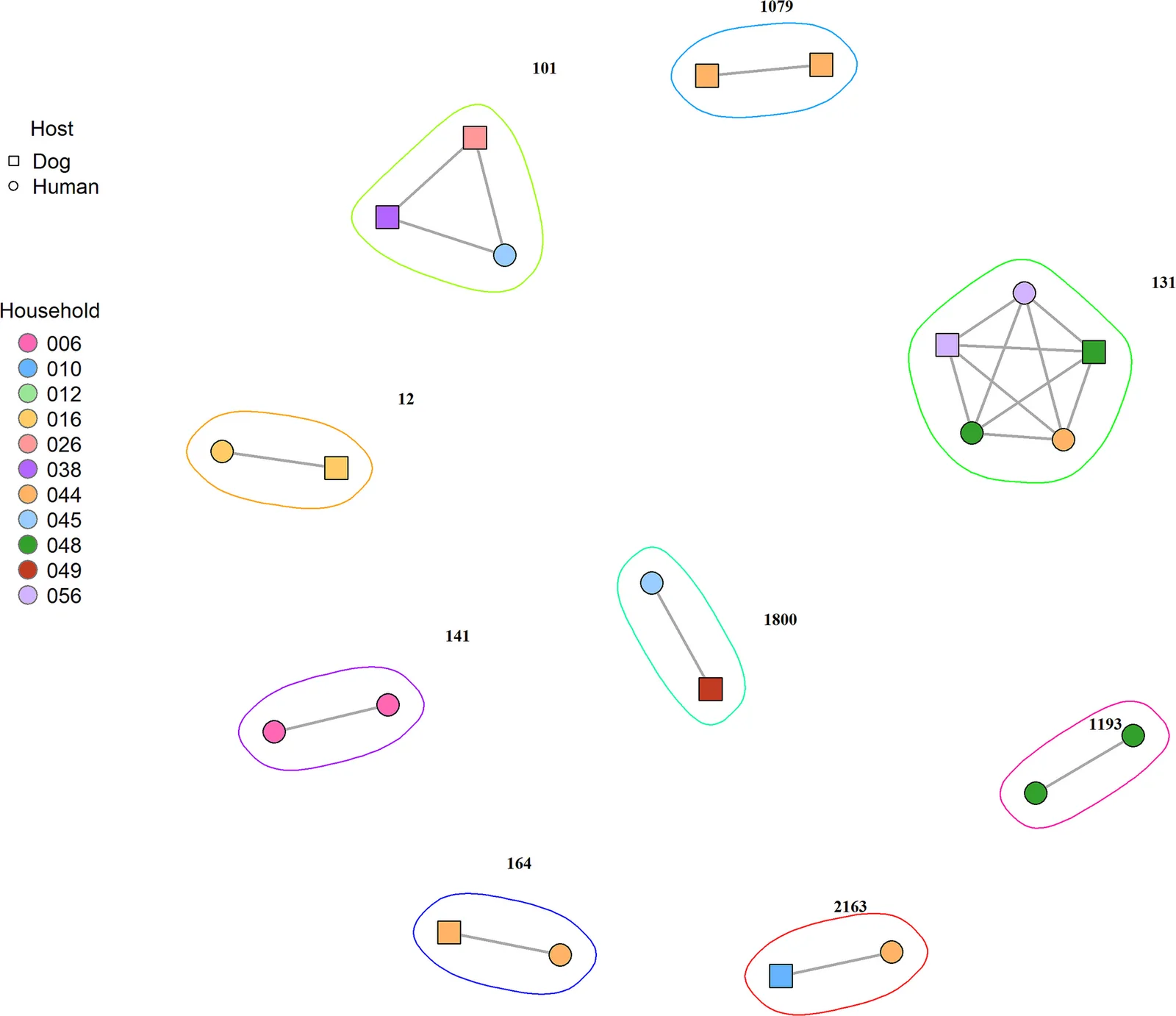
Separated networks of clonal *E. coli* present in different individuals isolated from humans and companion dogs from 24 households. The clonal *E. coli* co-occurrence between humans (circles) and companion dogs (squares) are indicated by nodes linked by grey lines if there was a five or fewer single nucleotide polymorphism difference between genomes. Nodes are coloured by the household and grouped by sequence type (ST)

*E. coli* genomes were investigated for putative clonal *E. coli* co-occurrences between individuals and comparison of AMR. Using the UKHSA five SNP threshold for clonal outbreak *E. coli*, there were 20 co-occurrences between different individuals. Seven were within the same household. Of these seven, four co-occurrences in HH044 (ST164), HH016 (ST12), HH056 (ST131) and HH048 (ST131) were between different host species. Two co-occurrences in different humans in the same household were observed in HH006 (ST141) and HH048 (ST1193). One co-occurrence between two dogs in the same household was observed in HH044 (ST1079). There were 13 instances of between-household co-occurrences with eight of these between different host species (Fig. 4).

ST1193-O75:H5 with the *fimH*64 allele co-occurred between two humans in HH048 and harboured 11 identical AMR determinants which were classified into seven different AMR classes (Supplementary Table S8). ST131-O16:H5 with the *fimH*41 allele co-occurred in humans (*n* = 2) and dogs (*n* = 3) across HH048, HH056, and HH044. These ST131-O16:H5 harboured three identical AMR determinants which were classified into three AMR classes (Supplementary Table S8).

**Fig. 5:**
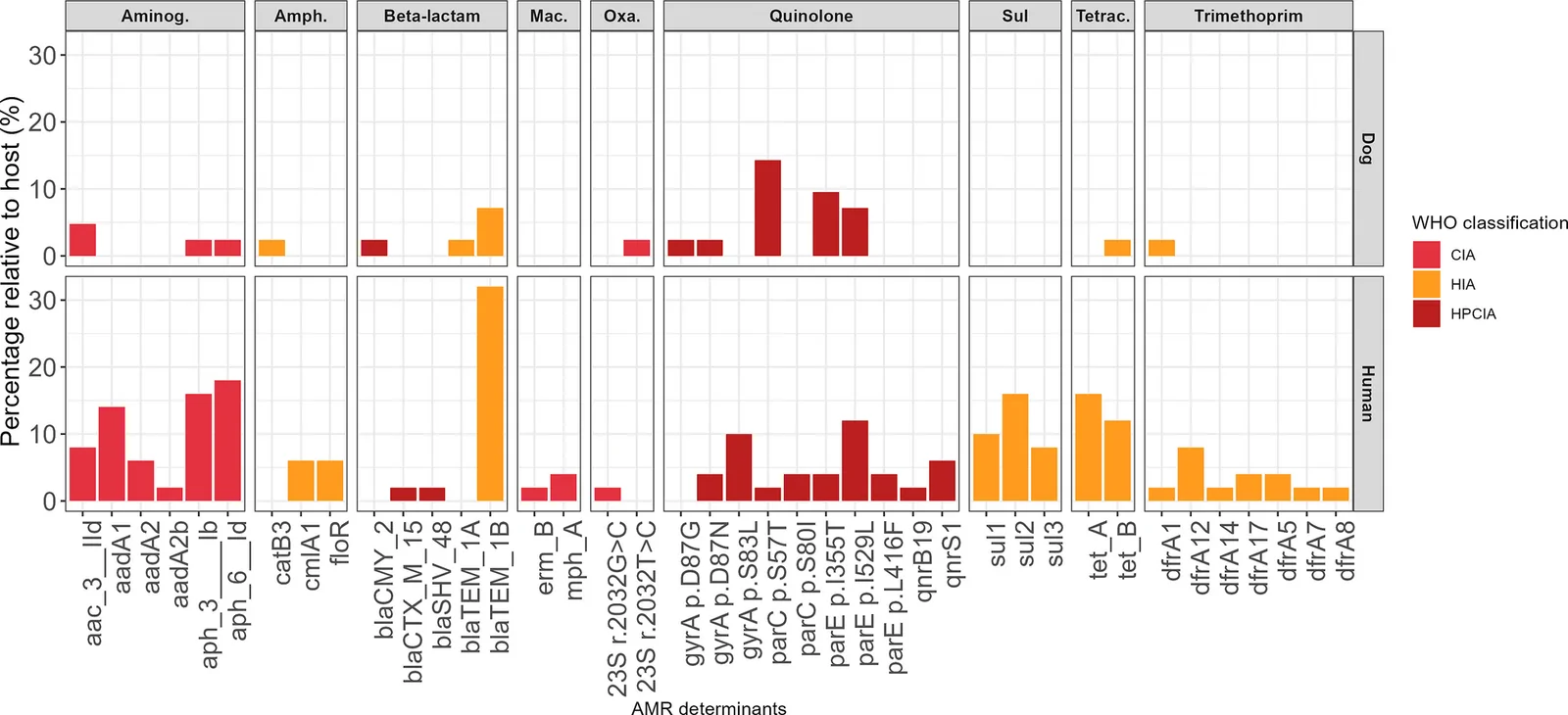
The distribution and percentage of antimicrobial resistance (AMR) determinants across 92 de-replicated *E. coli* genomes on the basis of the same ST, same AMR genotype, and found within the same sample. AMR determinants are classified into their respective antimicrobial class: Aminoglycosides (Aminog.), Amphenicols (Amph.), Beta-lactams, Oxazolidinones (Oxa.), Macrolides, Quinolones, Sulphonamides, Tetracyclines (Tetrac.), and Trimethoprim. Antimicrobial classes are classified as Highly Important (HIA), Critically Important (CIA), and Highest Priority Critically Important (HPCIA) according to the WHO Medically Important Antimicrobials List (WHO MIA List). *E. coli* from humans and companion dogs are separated

Across the 92 de-replicated genomes (Supplementary Table S9), the aminoglycoside resistance group was the most abundant AMR determinant class in *E. coli* isolated from human samples (*n* = 36/92) and the quinolone resistance group conferred by point mutations was the most abundant AMR determinant in dogs (*n* = 15/92) (Fig. 5). *E. coli* from human samples harboured more unique AMR determinants (median = 1, IQR = 3) than *E. coli* isolated from dog samples (median = 0, IQR = 1). A Wilcoxon rank-sum test showed a statistically significant difference in the number of unique AMR determinants between *E. coli* isolated from humans and dogs (*p* = 0.001).

## Discussion

We have assessed *E. coli* co-occurrence between humans and companion dogs, and AMR determinants by investigating the general *E. coli* population without selective enrichment for AMR. As this study was cross-sectional, this was assessed by the presence of similar *E. coli* co-occurring in different hosts. Putative clonal co-occurrences may differ in virulence factor content, even when genome-wide similarity is high. On this basis, we identified limited within-household clonal *E. coli* co-occurrences between humans and their companion pets.

The limited within-household cross-species co-occurrences in our study (4/24 households) aligns with previous pulse-field gel electrophoresis (PFGE) approaches, which showed cross-species co-occurrences within 4–9.8% of households (4%, 1/25 households; 8.8%, 3/34 households; 9.8%, 6/61 households) [30, 55, 78]. This work also aligns with limited cross-species extended-spectrum beta-lactamase (ESBL)/AmpC beta-lactamase-producing *E. coli* co-occurrence that was also observed using higher resolution WGS investigations by Toombs-Ruane et al. [84]. Longitudinal WGS work by Menezes et al*.* identified two animal-human pairs (2/59 households) with co-occurring ESBL-producing *E. coli*, where one household showing long-term carriage within the dog and subsequent presence within the co-habiting human, which would indicate stronger evidence of transmission [50]. Within this study, there were also 13 co-occurrences between households, which may suggest a common external source with which the hosts in this study may interact leading to clonal *E. coli* co-occurrence. By applying WGS with a 5-SNP threshold and analysing *E. coli* independent of AMR, our work was able to quantify the diversity of this bacterium within human and dog hosts and demonstrate a limited number of co-occurrences within and between households.

The co-occurrence of *E. coli* within pet dogs and owners can be reduced by exercising personal hygiene like washing hands after handling animals or their environment, using cleaning gloves when cleaning up after the pet, and limiting physical contact [79]. Shared contaminated environments within veterinarian settings, as well as the period of time exposed to contaminated spaces can contribute to bacterial transmission [81]. Practicing proper hand hygiene in combination with adequate sanitation, cleaning or disinfection of environmental surfaces can prevent the transmission of AMR bacteria within households [48].

The GWAS identified that the type-F conjugative transfer system pilin assembly thiol-disulphide isomerase (*trbB*) gene was associated with *E. coli* isolated from humans in this dataset. This gene is involved with the production of an F-pilus, which is used for conjugation and the transport of plasmid DNA between Gram-negative bacteria [7, 18, 58]. The conjugation process is central for the spread and distribution of mobile AMR genes, and can be facilitated by F-pili [58]. This human-associated gene was identified across multiple different phylogroups and identified on plasmid contigs within this dataset. However, the work in this study does not show absolute host specificity; to identify potential *trbB* human association more generally, further work with larger datasets from humans and dogs from diverse locations is needed to validate the results.

The combined unitig and gene presence and absence approach has been used in a previous *E. coli* GWAS study which has highlighted potential genetic determinants that may contribute to host specificity [83]. Both the unitig and the gene presence and absence approach were used as they capture both the associated gene and any associated variation within the gene. The gene presence and absence approach identifies associated genes which may have been lost or acquired by the *E. coli*, whereas the unitig approach offers higher resolution for capturing SNP variants within genes and extends to non-coding regions. Overlaps between approaches indicate a robust association at both the gene and the SNP level. An association using both approaches would complement and strengthen an observation that there was a significant association in this dataset.

The association of *trbB* in *E. coli* from humans, where AMR presence was more prevalent in this study, suggests that further investigation is needed to determine whether or not this could be a potential marker for the presence of AMR determinants in human-associated strains. Long-read sequencing confirmed the *trbB* gene linkage to AMR genes and regions associated with insertion sequences. This is consistent with previous work reporting that F-plasmids containing the *trbB* gene have co-occurred with plasmid-mediated AMR determinants [13, 90]. While our study indicates co-localisation of the *trbB* gene and AMR genes co-occurred in *E. coli* from humans, further work is still required to assess if it could be a reliable marker for the presence of AMR.

High-risk clones are defined as globally spread, possessing various AMR determinants, with abilities to persist and colonise a host for at least six months, spread between hosts, show increased pathogenicity, and cause severe/recurring infections [61]. However, despite ST131-O16:H5 and ST1193-O75:H5 being putative MDR and having been identified in previous literature as lineages of concern [61], it is unknown whether or not these *E. coli* isolates have the capacity to colonise a host for a prolonged period and cause severe/recurring infections. The ST131-O16:H5 with the *fimH*41 allele lineage identified in this dataset co-occurred across species and between households, falls within the previously described ST131-A clade [49], and has been identified in both human clinical cases and companion animals [21, 40]. In addition, ST1193-O75:H5 with the *fimH*64 allele has been highlighted as an emerging globally disseminated MDR ST [33, 86]. Our findings show that high-risk ST131-O16:H5 and ST1193-O75:H5 clones were detected in both humans and companion animals within the same household setting. This suggests that both owners and pets may serve as reservoirs of ST131-O16:H5 and ST1193-O75:H5 clones. This is consistent with previous work where an MDR and virulent *E. coli* subclone carriage was identified in human populations [65], and the subsequent emergence of the same MDR and virulent *E. coli* clonal group identified in companion animals [8]. If surveillance for high-risk clones were required, all hosts within a household should be taken into account.

AMR *E. coli* poses a serious health threat when treating infections as AMR can lead to less effective treatments, a delay in appropriate antimicrobial treatment, and increases in mortality for extra-intestinal bloodstream infections in both humans and companion pets [3, 24, 67]. In this study, the *E. coli* isolated from humans harboured more genes conferring resistance to different AMR drug classes and more AMR determinants overall than from dogs. Our findings align with previous results investigating human and canine faeces in urban environments, where a larger proportion of human isolates were phenotypically resistant to trimethoprim-sulfamethoxazole, ciprofloxacin, and third- and fourth-generation cephalosporins [89]. This is also observed in veterinary settings, where *E. coli* from veterinarians had higher rates of resistances to multiple antibiotics than *E. coli* from dogs [14]. Within the work in this study, it is not possible to definitively say that AMR present in both hosts would be a result of transmission as AMR *E. coli* could also be acquired through environmental exposure [62]. The prevalence of AMR *E. coli* in both humans and dogs more importantly highlights the concern that both species within the same household can harbour AMR *E. coli* and aid in the possible dissemination of AMR.

Virulence gene analysis revealed no *stx* genes in this collection, while the *eae* fimbriae gene was identified in two *E. coli* genomes from the same dog sample. However, the samples in this work containing putative ExPEC were from different households and none of the *E. coli* that co-occurred between human–human pairs and human–dog pairs from the same household were classified as putative ExPEC, suggesting minimal observed ExPEC risk in this study.

Our cross-sectional work does not directly demonstrate transmission or sharing as this would require a multi-sample longitudinal investigation. However, this cross-sectional study suggests there was limited inter-species *E. coli* co-occurrence. There is also a possibility of *E. coli* acquisition via a shared external source. This exposure could be through exposure to shared environmental contamination like veterinary clinics or dog parks, where *E. coli* and AMR can be shed by multiple dogs [62]. Additionally, households may be exposed to the same contaminated food [28, 52, 71]. Spatial proximity between households was not assessed in this study, but could be incorporated in future studies to assess potential transmission links. It is also important to note that while limited co-occurrence and thus potential sharing of *E. coli* was identified, our approach used convenience-sampling where we examined a single timepoint within each household and only isolated up to eight *E. coli* per sample. Previous work using a lower resolution Randomly Amplified Polymorphic DNA (RAPD) argues that a practical approach for assessing *E. coli* lineages in normal human enteric microbiota are 10 colonies per sample [53]. To investigate potential transmission further, larger datasets involving more households and larger numbers of *E. coli* isolates from each sample are necessary. This is supported by previous studies where, when up to 20 *E. coli* isolates were taken, 17% of human–dog pairs (19/115 within-household human–dog pairs) had identical *E. coli* PFGE co-occurrences [38].

The *E. coli* faecal sample recovery rate within this study may be affected by the freeze–thaw process during long-term storage. This freeze–thaw process may injure bacterial cells and reduce the number of culturable *E. coli* [82]. Moreover, the TBX agar growth medium used cannot detect around 5% of *E. coli* that are glucuronidase negative [29], including *E. coli* O157 [64]. This may limit our observations on the diversity of *E. coli* within these faecal samples. Overall, *E. coli* carriage rates are variable, ranging from 88.0%—95% in humans [1, 4]. When comparing different culture-approach studies in healthy dogs, *E. coli* presence in faeces and rectal samples have been found to vary between 10.9% to 100% [14, 39, 50, 68]. Differences in *E. coli* recovery rates will also be affected by differences in the stool sample collection, whether or not antimicrobials were used and the culture method.

## Conclusion

Our study indicated limited *E. coli* co-occurrence between humans and their pet dogs using a WGS approach without selective enrichment for AMR. This study also contributes to the body of evidence showing that clonal *E. coli* with potential to harbour AMR determinants can be present in both humans and their cohabiting animals. While the directionality of *E. coli* and AMR transmission between humans and pets cannot be inferred in this study, it is evident that both hosts can contribute to the overall dissemination and spread of clinically relevant *E. coli* clones of concern. It is important to take all hosts within the household unit into account when identifying and implementing interventions for reducing the spread of clinically important AMR *E. coli*.

Although co-occurrence within households was limited, the sharing of clonal *E. coli* between humans and dogs from different households indicates the complexity of potential transmission between hosts outside of the home. While the direction of transmission between hosts can never be inferred from a single time point study, it is clear that the co-occurrence of *E. coli* between humans and dogs within households occurred at a limited frequency. However, the results indicated that other routes of transmission could be equally important and should be investigated, highlighting the complexity of potential transmission between hosts in wider society.

## Supplementary Information

Supplementary Material 1.

Supplementary Material 2.

## Data Availability

The sequence data supporting the conclusions of this article are available in the European Nucleotide Archive under the accession ID PRJEB104018. The dataset supporting the conclusions of this article are included within the article, the supplementary document, and the Excel spreadsheet.

## Abbreviations

WGS: Whole genome sequencing
MLST: Multilocus Sequence Type
ST: Sequence Type
SNP: Single Nucleotide Polymorphism
HGT: Horizontal Gene Transfer
AMR: Antimicrobial Resistance
MDR: Multidrug-Resistance
ExPEC: Extraintestinal Pathogenic *E. coli*
BPW: Buffered Peptone Water
MAC: MacConkey
HPC: High-Performance Computing
UKHSA: United Kingdom Health Security Agency
WHO: World Health Organization
PFGE: Pulse Field Gel Electrophoresis
RMF: Raw Meat Fed
ESBL: Extended Spectrum Beta Lactamase
GWAS: Genome-Wide Association Study
