# Supplementary material for: Whole genome comparative analysis shows limited Escherichia coli co-occurrence within co-habiting humans and dogs
Source: BMC Genomics. 2026 Aug 4;27:900. doi: 10.1186/s12864-026-13236-2 (PMC13721559; doi:10.1186/s12864-026-13236-2)
Supplement: Supplementary file 1 [file 12864_2026_13236_MOESM1_ESM.docx]

Supplementary:

**Supplementary methods S1: Sample collection and processing**

Human and animal faecal samples were collected by owners using instructions provided by the RVC. Human faeces were collected in sterile ‘FeCol’ collection papers (Alpha Laboratories Ltd, UK) and transferred into sterile plastic containers, or directly into sterile plastic containers. Partial dog faeces that did not touch the ground were transferred into sterile plastic containers.

**Supplementary methods S2: *E. coli* colony isolation**

Up to four colonies of each distinct morphology on a plate were taken (maximum of eight colonies). The typical *E. coli* morphology would be a clear circular convex blue/green coloured colony. Morphologies that consistently exhibited other characteristics: a less vibrant but still blue/green sheen, a more mucoid colony with a blue/green sheen, or a non-circular colony with a blue/green sheen, were also chosen.

**Supplementary methods S3: Trimming and mean read depth**

The raw paired-end reads were trimmed using the -phred33 and ILLUMINACLIP:[library adapter]:2:30:10 LEADING:3 TRAILING:3 SLIDINGWINDOW:4:25 MINLEN:36 parameters.

To assess the mean read depth, trimmed reads were aligned to the genome assemblies using the Burrows-Wheeler aligner v.0.7.17 (1), and analysing the read depth and coverage using samtools v1.9 (2) and bcftools v.18 by SAMtools (2) using default parameters.

**Supplementary methods S4: Reference genome selection**

The reference that was chosen for each ST was defined as: 1) A reference identified by Referenceseeker for at least half of the genomes within that ST, 2) the reference with the highest average nucleotide identity, and 3) lowest average MASH distance.

Reads from each ST of interest were aligned to their respective reference whole genome assembly (ST10 reference (Genbank Accession assembly ID: GCF_900636145.1), ST101 reference (Genbank Accession assembly ID: GCF_001900815.1), ST1079 reference (Genbank Accession assembly ID: GCF_000468515.1), ST1193 reference (Genbank Accession assembly ID: GCF_019443685.1), ST12 reference (Genbank Accession assembly ID: GCF_010706435.1), ST131 reference (Genbank Accession assembly ID: GCF_026651285.1), ST141 reference (Genbank Accession assembly ID: GCF_016889685.1), ST164 reference (Genbank Accession assembly ID: GCF_020683245.1), ST1800 (Genbank Accession assembly ID: GCF_020683145.1), ST2163 reference (Genbank Accession assembly ID: GCF_904842665.1), and ST372 reference (Genbank Accession assembly ID: GCF_023658365.1)).

**Supplementary methods S5:**

*E. coli* genomes containing the human-associated unitig on plasmid-associated contigs were selected for long-read sequencing. Libraries were formed from genome extractions using the Native barcoding kit and were sequenced on a MinION (Oxford Nanopore Technologies, Oxford, UK). Filtlong v0.2.0 (<https://github.com/rrwick/Filtlong>) was used to trim long reads using a 1000 bp cut-off. Long read sequencing data were deposited in the European Nucleotide Archive under the accession ID PRJEB104018.

Long reads were assembled using Canu v2.2 (1), Flye v2.9 (2) and Raven v1.1.10 (3). Medaka v1.4.3 (<https://github.com/nanoporetech/medaka>) was used to align long reads to these assemblies polish them. To create hybrid assemblies utilising the trimmed long and short reads, Unicycler v0.4.8 (4) was used. To correct for mismatches, short reads were aligned to the assemblies using BWA v0.7.10 (5). Hybrid assemblies were corrected using Pilon v1.22 (6), and five rounds was chosen as further implementation provides minimal additional error corrections (7) (Supplementary Figure 1A).

Socru v2.2.4 (8) was used to determine the orientation of the ribosomal operons within the chromosome. ART v2.5.8 (9) was used to simulate paired Illumina reads from the assemblies. ARIBA v2.14.6 (10) was used to search the Illumina and simulated reads against the ResFinder (11) and PlasmidFinder (12) databases. Abricate v1.0.1 (<https://github.com/tseemann/abricate>) was used to help investigate any discrepant ARIBA results between the long/hybrid assemblies and the short reads. BWA was used to align trimmed Illumina reads to the assemblies, and samtools v1.9 and bcftools v1.8 (13) were used to identify variants between these reads and the assemblies. Assemblies that consisted of circularised plasmid contigs, had plausible ribosomal operon arrangements, and were not missing any AMR genes or plasmid replicons were selected for further analysis. In cases where multiple assemblies met these criteria for an isolate, the assembly with the fewest variants with Illumina reads was used (Supplementary Figure 1B; Supplementary Table S1).

Abricate was used to determine the location of AMR genes and plasmid replicons, and BLASTn [11] was used to identify the location of the *trbB* gene (NZ_AIAG01000123.1). 90% identity and 90% coverage cut-offs were used. ISEScan v1.7.3 (14) was used to determine the location of “complete” insertion sequences (ISs).

Long/hybrid assemblies were annotated using BAKTA v1.8.2 (15) and genes were clustered to form a dendrogram based on the presence-absence matrix using Roary v3.10.2 (16) and a 95% identity cut-off. EasyFig v2.1 (17) was used to align the plasmid sequences (Supplementary Figure 5).

**Supplementary results R1:**

In the nine long-read sequenced *E. coli* genomes, the *trbB* gene was found within plasmid sequences containing the IncFIB(AP001918)_1 plasmid replicon. Eight of these plasmid sequences also contained the IncFII(29)_1_pUTI89 plasmid replicon and six contained the Col156_1 plasmid replicon (Supplementary Figure 5).

The nine long-read assembled genomes contained the AMR gene, *mdf(A)*, in their chromosome. Additional AMR genes were found in the *trbB*-containing plasmid sequences. The *trbB*-containing plasmids contained 0-3 AMR genes and these AMR genes were found in close proximity to insertion sequences in a specific region of this plasmid.

**Supplementary Figures**

Supplementary Figure 1: Long read sequencing workflow (A) and quality control selection flowchart (B) for *E. coli* genomes containing the human-associated unitig on plasmid-associated contigs.

Supplementary Figure 2: Phylonium midpoint rooted phylogenetic tree of the collection of 217 *E. coli* genomes isolated from humans and their companion dogs from 24 households, with a tree scale estimating 0.003 nucleotide substitutions per site. The host species (left column), phylogroup (middle column) and household (right column) are plotted against the tree. The node colours represent bootstrap support values.

Supplementary Figure 3: Venn diagram of all multilocus sequence types from the 217 *E. coli* genomes isolated humans and their companion dogs from 24 households, separated by host species.

Supplementary Figure 4: Phylonium midpoint rooted phylogenetic tree of the collection of 217 *E. coli* genomes isolated humans and their companion dogs from 24 households, with a tree scale estimating 0.003 nucleotide substitutions per site. The host species (left column), phylogroup (right column) are labelled. Genomes with the human associated hypothetical protein encoding gene, group 4813 (*trbB*), are identified by red dots at the end of the tree tips.

Supplementary Figure 5. Alignment of the *trbB*-containing plasmid sequences with AMR genes coloured red and insertion sequences coloured purple from the nine long-read sequenced *E. coli* genomes, along with a dendrogram based on a gene presence-absence matrix of the plasmids, coloured by source, household, sample, ST and the presence of plasmid replicons.

**References**

1.Koren S, Walenz BP, Berlin K, Miller JR, Bergman NH, Phillippy AM. Canu: scalable and accurate long-read assembly via adaptive k-mer weighting and repeat separation. Genome Res. 2017;27(5):722-36.

2.Kolmogorov M, Yuan J, Lin Y, Pevzner PA. Assembly of long, error-prone reads using repeat graphs. Nature Biotechnology. 2019;37(5):540-6.

3.Vaser R, Šikić M. Time- and memory-efficient genome assembly with Raven. Nat Comput Sci. 2021;1(5):332-6.

4.Wick RR, Judd LM, Gorrie CL, Holt KE. Unicycler: Resolving bacterial genome assemblies from short and long sequencing reads. PLoS Computational Biology. 2017;13(6):e1005595.

5.Li H, Durbin R. Fast and accurate long-read alignment with Burrows-Wheeler transform. Bioinformatics. 2010;26(5):589-95.

6.Walker BJ, Abeel T, Shea T, Priest M, Abouelliel A, Sakthikumar S, Cuomo CA, Zeng Q, Wortman J, Young SK, Earl AM. Pilon: An Integrated Tool for Comprehensive Microbial Variant Detection and Genome Assembly Improvement. PLOS ONE. 2014;9(11):e112963.

7.Luan T, Commichaux S, Hoffmann M, Jayeola V, Jang JH, Pop M, Rand H, Luo Y. Benchmarking short and long read polishing tools for nanopore assemblies: achieving near-perfect genomes for outbreak isolates. BMC Genomics. 2024;25(1):679.

8.Page AJ, Ainsworth EV, Langridge GC. socru: typing of genome-level order and orientation around ribosomal operons in bacteria. Microbial Genomics. 2020;6(7).

9.Huang W, Li L, Myers JR, Marth GT. ART: a next-generation sequencing read simulator. Bioinformatics. 2012;28(4):593-4.

10.Hunt M, Mather AE, Sanchez-Buso L, Page AJ, Parkhill J, Keane JA, Harris SR. ARIBA: rapid antimicrobial resistance genotyping directly from sequencing reads. Microbial Genomics. 2017;3(10):e000131.

11.Bortolaia V, Kaas RS, Ruppe E, Roberts MC, Schwarz S, Cattoir V, Philippon A, Allesoe RL, Rebelo AR, Florensa AF, Fagelhauer L, Chakraborty T, Neumann B, Werner G, Bender JK, Stingl K, Nguyen M, Coppens J, Xavier BB, Malhotra-Kumar S, Westh H, Pinholt M, Anjum MF, Duggett NA, Kempf I, Nykasenoja S, Olkkola S, Wieczorek K, Amaro A, Clemente L, Mossong J, Losch S, Ragimbeau C, Lund O, Aarestrup FM. ResFinder 4.0 for predictions of phenotypes from genotypes. Journal of Antimicrobial Chemotherapy. 2020;75(12):3491-500.

12.Carattoli A, Hasman H. PlasmidFinder and *in silico* pMLST: identification and typing of plasmid replicons in whole-genome sequencing (WGS). de la Cruz F, editor. New York, NY: Springer US; 2020. 285-94 p.

13.Danecek P, Bonfield JK, Liddle J, Marshall J, Ohan V, Pollard MO, Whitwham A, Keane T, McCarthy SA, Davies RM, Li H. Twelve years of SAMtools and BCFtools. GigaScience. 2021;10(2):giab008.

14.Xie Z, Tang H. ISEScan: automated identification of insertion sequence elements in prokaryotic genomes. Bioinformatics. 2017;33(21):3340-7.

15.Schwengers O, Jelonek L, Dieckmann MA, Beyvers S, Blom J, Goesmann A. Bakta: rapid and standardized annotation of bacterial genomes via alignment-free sequence identification. Microbial Genomics. 2021;7(11).

16.Page AJ, Cummins CA, Hunt M, Wong VK, Reuter S, Holden MTG, Fookes M, Falush D, Keane JA, Parkhill J. Roary: rapid large-scale prokaryote pan genome analysis. Bioinformatics. 2015;31(22):3691-3.

17.Sullivan MJ, Petty NK, Beatson SA. Easyfig: a genome comparison visualizer. Bioinformatics. 2011;27(7):1009-10.
